# A Review of Nuclear Imaging in Takotsubo Cardiomyopathy

**DOI:** 10.3390/life12101476

**Published:** 2022-09-23

**Authors:** Jemimah Nayar, Kevin John, Anil Philip, Lina George, Anu George, Amos Lal, Ajay Mishra

**Affiliations:** 1 Department of Nuclear Medicine, Christian Medical College, Vellore 632004, India; 2 Department of Medicine, Tufts Medical Center and Tufts University School of Medicine, Boston, MA 02111, USA; 3 Department of Medicine, Kuriakose Chavara Memorial Hospital, Noornad 690571, India; 4 Department of Pulmonary Medicine, Kuriakose Chavara Memorial Hospital, Noornad 690571, India; 5 Department of Internal Medicine, Saint Vincent Hospital, Worcester, MA 01608, USA; 6 Multidisciplinary Epidemiology and Translational Research in Intensive Care Group, Department of Medicine, Division of Pulmonary and Critical Care Medicine, Mayo Clinic, Rochester, MN 55905, USA; 7 Department of Cardiology, Saint Vincent Hospital, Worcester, MA 01608, USA

**Keywords:** Takotsubo cardiomyopathy, nuclear imaging, nuclear cardiology

## Abstract

Takotsubo cardiomyopathy or Takotsubo Syndrome (TTS) is a reversible left ventricular dysfunction syndrome that is increasingly being recognized. Recent advances in nuclear imaging have allowed us to study TTS in greater detail. We searched the PubMed and Medline databases and identified 53 publications with 221 patients reporting nuclear imaging findings in TTS. The age of the patients ranged from 17 to 87 years and were predominantly women (88.2%). The TTS variant was apical (typical) in 170 (76.9%), mid-ventricular in 23 (10.4%), and basal (reverse TTS) in 2 (0.9%). Cardiac perfusion was assessed using ^99m^Tc sestamibi (MIBI) SPECT, ^99m^Tc tetrofosmin SPECT, ^201^Tl SPECT, ^82^Rb PET, ^201^Tl SPECT, and ^13^N ammonia PET. Additional studies used were ^123^I MIBG SPECT, ^123^I BMIPP SPECT, ^18^F FDG PET, ^67^Ga citrate, and ^11^C hydroxy-ephedrine. A perfusion defect was seen in 69 (31.2%), and an inverse perfusion–metabolism mismatch (normal or near-normal perfusion with absent myocardial metabolic activity) was seen in 183 (82.8%) patients. Nuclear imaging has a significant role in evaluating, diagnosing, and prognosticating patients with TTS. As nuclear imaging technology evolves, we will surely gain more insights into this fascinating disorder.

## 1. Introduction

Takotsubo cardiomyopathy, or Takotsubo Syndrome (TTS), is a reversible left ventricular dysfunction syndrome that was described over two decades ago. In its most common variant, the left ventricle (LV) resembles a Japanese octopus (tako) trapping pot (tsubo), from which it gets the name. Although most of the initial reports of TTS were from Japan, it is now reported in almost every country. It is estimated that 1–2% of all patients presenting with acute coronary syndrome have TTS [1].

TTS is a transient cardiac syndrome, and patients present with chest pain, EKG abnormalities, and elevated cardiac enzymes, often in the setting of acute stress. Although TTS is reversible, complications such as arrhythmia, pump failure, outflow tract obstruction, cardiac rupture, and embolization can cause morbidity and mortality. The Revised Mayo Clinic Criteria is widely used for diagnosing TTS and requires the presence of transient LV systolic dysfunction, absence of obstructive coronary disease, new EKG abnormalities or modest elevation in troponin, and absence of pheochromocytoma or myocarditis [1]. Although the mechanisms underlying this syndrome are not fully known, a link to sudden stressors and high-catecholamine states has been established.

Over the last decade, nuclear imaging techniques have seen tremendous development and application. Nuclear imaging techniques have allowed us to study in detail several diseases, including TTS, to appreciate its pathophysiology, improve diagnostic accuracy, and provide prognostic information. This narrative review aims to summarize the current literature on the use of nuclear imaging in TTS.

## 2. Materials and Methods

We searched the PubMed and Medline databases to identify studies. Search terms included “Takotsubo Cardiomyopathy (MeSH term)”, “nuclear imaging,” and names of individual tracer compounds. References of manuscripts from the initial search were used to find additional manuscripts. Studies published in English, including adults with TTS who underwent nuclear imaging, were eligible to be included in this review. We searched the listed databases from inception to May 2022. Articles that did not have the patient’s details, opinions, comments, letters, and articles not published in English were excluded from the analysis. Two independent clinicians reviewed all articles.

## 3. Results

**Figure 1:**
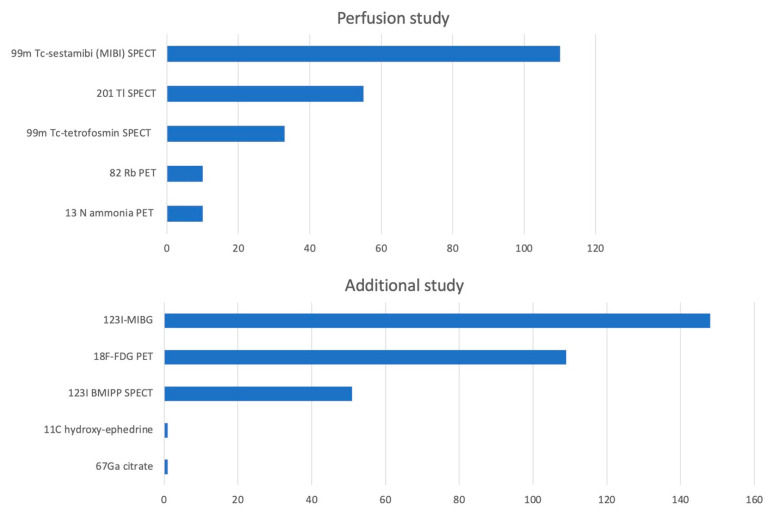
Common tracer compounds used for nuclear imaging in Takotsubo cardiomyopathy.

From a total of 5590 papers that were assessed for eligibility, we excluded 888 comments, 161 editorials, 1439 letters, 656 reviews, and 2 guidelines. We excluded an additional 2391 papers which did not have data on nuclear imaging. A total of 53 papers with 221 patients were included in the final review (Table 1). The age of the patients ranged from 17 to 87 years and were predominantly women (88.2%). The TTS variant was apical (typical) in 170 (76.9%), mid-ventricular in 23 (10.4%), and basal (reverse TTS) in 2 (0.9%). Most patients had a single region of involvement. Cardiac perfusion was assessed at admission for all patients using ^99m^Tc sestamibi (MIBI) SPECT, ^99m^Tc tetrofosmin SPECT, ^201^Tl SPECT, ^82^Rb PET, and ^13^N ammonia PET (Figure 1). Additional studies employed were ^123^I MIBG SPECT, ^123^I BMIPP SPECT, ^18^F FDG PET, ^67^Ga citrate, and ^11^C hydroxy-ephedrine. The most common studies performed in each category were ^99m^Tc sestamibi (MIBI) SPECT and ^123^I MIBG SPECT, respectively. The tracers used for perfusion and additional studies depended on the facility’s choice. A perfusion defect was seen in 69 (31.2%), and an inverse perfusion–metabolism mismatch (normal or near-normal perfusion with absent myocardial metabolic activity) was seen in 183 (82.8%) patients. No adverse events were reported due to radiotracer injection.

## 4. Discussion

Takotsubo cardiomyopathy, or Takotsubo syndrome (TTS), was first described in Japan in 1990 and was so named because of the resemblance of the heart to a traditional Japanese octopus trapping pot. Since its initial description, the syndrome has been diagnosed worldwide in several settings, including the intensive care unit. With the COVID-19 pandemic, Takotsubo cardiomyopathy has been described both as a complication of COVID-19 infection and as a consequence of the emotional stress associated with social distancing [53]. It is estimated that 1–2% of all patients presenting with an initial diagnosis of acute coronary syndrome have TTS [1]. With further medical advances, particularly in diagnostic cardiology, the recognition and hence the incidence of TTS is bound to increase.

With the turn of the century, nuclear imaging has seen rapid advances and increased application in several disciplines. With our ability to engineer targeted tracer compounds and improvement in imaging techniques, we can study the pathogenesis of several diseases in detail, including TTS. Various nuclear imaging techniques have been used to study TTS, including perfusion and metabolic studies. Not only are we able to obtain a global understanding of cardiac metabolic patterns, but also granular patterns including the uptake of individual metabolites such as glucose and fatty acids. We can also image the sympathetic innervation of the heart and identify defects in the same. With all these techniques, we now have a better understanding of TTS. To the best of our knowledge, this is the most exhaustive review of nuclear imaging in TTS to date, with 221 patients. We aim to provide a comprehensive update on the application of nuclear imaging in TTS, the new insights gained on its pathophysiology, and possible applications of this technology in the future.

The pathophysiology of TTS is not fully understood, but several postulated hypotheses exist. It is reasonably clear that there is a relationship between TTS and increased levels of stress and catecholamine levels. There are reports of increased incidence of TTS after natural calamities. One such observation was made during the Niigata earthquake when the incidence of TTS increased 24 times higher than baseline near the earthquake’s epicenter [54]. In the following paragraphs, we will discuss the evidence supporting the current hypothesis regarding its pathophysiology and observations suggesting that TTS may be more complicated and heterogeneous than previously thought.

### 4.1. The Role of Increased Sympathetic Drive

Increased sympathetic drive and catecholamine levels are central to the pathophysiology of TTS. Catecholamines induce an increase in intracellular Ca^2+^, which is thought the be the cause of myocardial damage [55]. Another contributing factor may be myocardial ischemia due to microvascular dysfunction. Catecholamine-induced oxygen deprivation and hypo-perfusion at onset could produce a long-term dysfunction of sympathetic neurons. Almost one-third of the cases we reviewed showed evidence of perfusion defect on nuclear imaging. Furthermore, the ‘inverse-mismatch pattern’ between perfusion and metabolism, which was seen in most cases, suggests that the metabolic dysfunction contributes more to the pathophysiology of TTS than hypo-perfusion itself. High epinephrine concentrations trigger a switch in intracellular signal trafficking in ventricular cardiomyocytes from stimulatory G_s_ protein to inhibitory G_i_ protein signaling via the β_2_-adrenoceptor. This, in turn, protects against the pro-apoptotic effects of intense β_1_-adrenoceptor activation [56]. However, this change also causes a negative inotropic effect.

In addition, it has also been assumed that the increased apical density and sensitivity of the β_2_ receptor to epinephrine may cause a prolonged downregulation of the β_2_ receptor and impaired uptake-1 function. This causes relatively high levels of epinephrine and norepinephrine in the synaptic cleft resulting in the slow recovery of these receptors and transporters compared to more basally located β_2_ receptors [57].

Differences in the regional distribution of cardiac innervation and local adrenoceptor density may be the major determinants behind the distinct patterns of TTS. In a canine model, β_2_-adrenergic receptor density increased from the basal toward the apical region [58]. The increased β-receptor density in the apical myocardium in post-menopausal women could explain the predominance of the apical pattern in this group. Similarly, the presentation of reverse TTS in younger patients may be due to the abundance of adrenoreceptors and sympathetic nerve endings at the base of the heart in this population [59].

### 4.2. Evaluation of Cardiac Sympathetic Innervation

^123^I-MIBG imaging (both planar and SPECT) is a vital tool to evaluate sympathetic innervation of the heart. Planar ^123^I-MIBG cardiac images are analyzed by computing the heart-to-mediastinal (H/M) ratio on both early and late images and the washout rate (WO) of the tracer from the myocardium between early and late acquisitions. In addition, regional tracer uptake is qualitatively assessed on SPECT images using a 5-point score in a 17-segment model. A reduced late H/M ratio (H/M_late_) and an increased WO are associated with a poor prognosis, whereas the role of SPECT analysis is still under investigation. In 2016, Christensen and colleagues demonstrated that ^123^I MIBG images in TTS showed a lower late (4-h) heart-to-mediastinum ratio (H/M_late_) (2.00 +/− 0.38) and a higher washout rate (WR) (45 +/− 12%) in the subacute state of TTC, both when compared with follow-up (H/M_late_: 2.42 +/− 0.45; *p* = 0.0004; WR: 33 +/− 14%; *p* = 0.0004) and when compared with the control group in the subacute state (H/M_late_: 2.34 +/− 0.60, *p* = 0.035; WR: 33 +/− 19%, *p* = 0.026) [60]. This difference did not persist during follow-up. Moreover, plasma epinephrine levels were elevated in patients with TTS at presentation and remained relatively elevated on follow-up compared to the control group. The low H/M_late_ and high WR on MIBG imaging in the acute state of TTS may be due to the blocking effect of high circulating levels of epinephrine on norepinephrine reuptake [60].

In a case of inverted TTS reported by Humbert et al., ^123^I-MIBG SPECT showed reduced uptake at the basal region, indicating a concordance between hypokinetic segment and region of decreased ^123^I-MIBG uptake [9]. Sestini and colleagues demonstrated adrenergic dysfunction at the apex in TTS patients that persisted even after apparent recovery [6]. At presentation, a reduced ^123^I-MIBG uptake was found in 21 patients (95%) on early and all patients on late images. This persisted in 50% of patients at three years of follow-up. Given that sympathetic fibers are more susceptible to hypoxia and acidosis, it is plausible that the initial insult destroys the sympathetic fibers before the myocardium is lost. This finding is significant because the denervated myocardium is supersensitive to catecholamines resulting in a higher risk of arrhythmia [61].

Assessing the sympathetic innervation with ^123^I-MIBG may have prognostic implications. Investigators from the Kindai University Hospital, Japan, retrospectively identified 90 patients with TTS who were divided into two groups based on the timing of LV function improvement: <1 month (S) group and >1 (L) month group [62]. The sympathetic nervous system activity assessed using ^123^I MIBG scintigraphy showed lower H/M_late_ (2.09 vs. 2.45, *p* = 0.01) and higher WO (33.9 vs. 26.4, *p* = 0.02) in the L group compared with the S group. There were more in-hospital complications in the L group (56% vs. 33.3%, *p* = 0.03), including higher rates of heart failure (45% vs. 23%, *p* = 0.03) and in-hospital death (8.0% vs. 0%, *p* = 0.03). These findings suggest that higher sympathetic nervous system activity is associated with a poor prognosis in patients with TTS, and ^123^I MIBG can be used as a risk stratification tool.

There may also be sex-dependent differences in the cardiac sympathetic outflow. Retrospective analysis of cardiac ^18^F Dihydroxyphenylalanine (DOPA) uptake in 133 patients showed significantly higher uptake in women (1.33 ± 0.21 vs. 1.18 ± 0.24, *p* < 0.001) [63]. The difference was most pronounced in the LV apex (1.30 ± 0.24 in women vs. 1.13 ± 0.25 in men, *p* < 0.001) and in individuals >55 years of age (1.39 ± 0.25 in women vs. 1.09 ± 0.24 in men, *p* < 0.001). Although this analysis was not carried out in patients with TTS, using ^123^I MIBG scintigraphy in this context provides clues as to why older women are more susceptible to TTS.

### 4.3. Role of Perfusion Abnormalities and Its Assessment in TTS

The role of perfusion abnormalities in TTS is a matter of debate as both increased and decreased perfusion have been described by radionucleotide perfusion studies (RPS). Anderson et al. analyzed the Intermountain Healthcare electronic medical records which contained 16 patients with TTS who had RPS [2]. The tracers used were ^82^Rb PET/CT in eight, ^99m^Tc sestamibi SPECT in six, and ^201^Tl SPECT in two. Perfusion defects were seen in 11 (68.7%) patients with TTS and commonly involved the apical or antero-septal-apical left ventricle, despite having patent coronaries. This proportion was much higher than our pooled data, identifying perfusion abnormalities in only one-third of the patients. It is also important to note that the defects do not correspond to any particular arterial territory—a defining feature of TTS that suggest microcirculatory dysfunction rather than epicardial coronary pathology.

While most studies demonstrated reduced perfusion in TTS by RPS, the results of a survey by Christensen et al. reported the opposite [64]. This group of investigators studied 25 patients with TTS using coronary angiography (CAG), echocardiography, cardiac magnetic resonance imaging (CMR), and ^13^N ammonia/^82^Rb PET in both the acute state and follow-up. In 17 patients, flow in the basal region was increased in the acute state (1.5 ± 0.1 vs. 1.2 ± 0.1 mL/g/minRPP−corrected, *p* < 0.01), whereas mid-ventricular (1.7 ± 0.1 vs. 1.6 ± 0.1 mL/g/minRPP−corrected, *p* = 0.21) and apical (1.4 ± 0.1 vs. 1.5 ± 0.1 mL/g/minRPP−corrected, *p* = 0.36) flow was unchanged between acute and follow-up, and within normal range. Similar findings were also reported in several case reports. (Table 1) It is plausible that the basal hyper-perfusion is a physiological compensation to meet the increased metabolic demand as basal contractility increases to compensate for the failing heart.

Quantifying the perfusion abnormality is important for prognostication, as demonstrated by Kobylecka et al. who used ^99m^Tc sestamibi gated SPECT/CT to assess myocardial perfusion [65]. Patients with a mean summed rest score (SRS) of four or more had lower left ventricular ejection fraction, perfusion defect size, total perfusion defect, number of akinetic segments in echocardiography, and number of segments with perfusion defect. The investigators also noted that the applied attenuation correction algorithms using CT images did not change the final result of myocardial perfusion imaging, indicating that the CT component of the SPECT/CT study is not required for TTS diagnosis. However, others with a different opinion support the use of hybrid cardiac SPECT/CT in TTS as it allows better assessment of coronary artery distribution and myocardial damage [66].

In summary, perfusion abnormalities may be a consequence of TTS rather than the primary cause. However, these studies highlight the role of microvascular dysfunction in the acute phase of TTS. Although not diagnostic, nuclear perfusion imaging does provide prognostic information in the acute phase of TTS.

### 4.4. Altered Myocardial Metabolism in TTS

Obunai and colleagues were among the earliest to describe altered myocardial metabolism in TTS [46]. In a case report published in 2005, they described profoundly reduced 18F-FDG PET uptake in the ballooned apical wall with relatively normal perfusion in TTS-a pattern similar to myocardial stunning. What is intriguing about this case, and many others reported later, was the degree of metabolic abnormality which is out of proportion to the perfusion defect—the so-called “reverse (or inverse) perfusion-metabolism mismatch” pattern [32,41,43]. Moreover, the metabolism of fatty acids was also disproportionately affected [67]. Notably, perfusion was reduced in many cases, despite having normal epicardial coronaries. However, the degree of the metabolic defect was almost always more significant than the perfusion abnormality.

Similar findings were observed in a prospective study of 18 patients by Kobylecka and colleagues [68]. No regional perfusion abnormalities were seen in 10 patients, while eight had mild perfusion defects. Even after prolonged fasting, heterogenous ^18^F FDG uptake was seen in 10 patients, and selective apical ^18^F FDG accumulation was seen in eight patients. In TTS, cardiomyocytes may switch from free fatty acid metabolism to anaerobic glycolysis after the initial insult. Altered metabolism at the LV apex was also appreciated when the total defect score (TDS) of ^123^I BMIPP and perfusion were semi-quantitatively determined using SPECT [12]. In this study which had 16 patients with TTS, ^123^I BMIPP abnormalities were exclusively observed in the apical area. As mentioned before, high epinephrine concentrations trigger a switch in intracellular signal trafficking in ventricular cardiomyocytes from stimulatory G_s_ protein to inhibitory G_i_ protein signaling. This may lead to downstream signaling that changes myocardial metabolism, perhaps as a protective mechanism. These changes can be detected and followed up with nuclear imaging studies.

In this context, the interaction of hyperglycemia and TTS also deserves mention. Its role in prognosis and infarct size has been evaluated in obstructive coronary artery disease and MINOCA [69]. Hyperglycemia may act as a metabolic trigger that unbalances the sympathetic response by altering signal transduction pathways. The HIGH-GLUCOTAKO investigators compared 28 TTS patients with hyperglycemia with 48 who were normoglycemic [70]. Norepinephrine levels along with ^123^I MIBG cardiac scintigraphy with late heart-to-mediastinum ratio (H/M_late_) and washout rate (WR) were used to assess sympathetic activity, in a subset of patients. The data showed a direct correlation between blood glucose levels and norepinephrine levels at admission (R^2^ = 0.39, *p* = 0.001). In the subset of TTS patients who underwent ^123^I MIBG cardiac scintigraphy, those with hyperglycemia had lower H/M_late_ values at both the acute phase (*p* < 0.001) and follow-up (*p* < 0.001). Reduction in blood glucose correlated well with changes in H/M_late_ after 24 months of follow-up (R^2^ = 0.38; *p* = 0.021). Significantly higher mortality (25 vs. 8.3%; *p* < 0.05) and HF events (21.4 vs. 8.3%; *p* = 0.001) were seen in the TTS group with hyperglycemia during follow-up.

### 4.5. Assessment of Neurobiological Activity

There is emerging evidence about the role of the neuro-humeral axis and the sympathetic stress response in cardiovascular disease. Suzuki et al. demonstrated significantly increased blood flow in the hippocampus, brainstem, and basal ganglia, and significantly decreased blood flow in the prefrontal cortex in the acute phase of TTS using 99m-Tc ethyl cysteinate dimer SPECT [71]. A longitudinal study of 293 patients showed that amygdalar activity (AmygA) measured by ^18^F FDG PET/CT had a significant bearing on the risk of cardiovascular disease events (standardized HR 1·59, 95% CI: 1·27–1·98; *p* < 0.0001) [72] A dysfunctional limbic system causing overactivation of the sympathetic nervous system leading to excessive catecholamine release is a proposed mechanism of TTS, which was studied using functional magnetic resonance imaging (fMRI) [73]. To test the hypothesis that heightened AmygA precedes the development of TTS and that those with the highest AmygA develop the syndrome earliest, Radfar et al. retrospectively identified 104 patients who had undergone ^18^F FDG PET/CT imaging (41 of whom subsequently developed TTS, and 63 matched controls) [74]. It was observed that patients who developed TTS had a higher baseline AmygA (*p* = 0.038), which also predicted TTS risk after adjusting for confounders (standardized hazard ratio: 1.643, 95% CI: 1.19–2.27; *p* = 0.003). Additionally, on average, individuals with the highest AmygA levels developed TTS two years earlier than those with lower levels (*p* = 0.028). Similar findings were reported in a prospective study conducted by independent investigators, albeit in a smaller sample size [75]. These findings provide strong evidence for the role of the neuro-humeral axis in TTS. Moreover, it may be possible to predict an individual’s susceptibility to developing TTS using nuclear imaging of the amygdala.

### 4.6. Knowledge Gaps in Our Understanding of TTS and New Questions from Nuclear Imaging Studies

While nuclear imaging studies have helped us better understand TTS’s pathophysiology, some observations raise more questions. There are several case reports of patients with TTS whose nuclear imaging findings contradict our current pathogenesis model. One such case is a 67-year-old heart-transplant patient who developed TTS [76]. The patient, who had heart transplantation for ischemic cardiomyopathy ten years prior, presented with heart failure. He received a definitive diagnosis of TTS after a comprehensive workup which included dual-isotope SPECT using ^201^Tl chloride and ^123^I BMIPP. Heart transplantation leads to extrinsic cardiac denervation due to surgical interruption of sympathetic and parasympathetic nerve fibers, and reinnervation may not always occur [77]. The extent of cardiac innervation after heart transplantation can be studied by early and late myocardial uptakes of MIBG, which reflects the distribution of the presynaptic nervous system and neuronal function. In this patient, the serial heart-to-mediastinal ratio level of ^123^I MIBG showed no significant increase on serial follow-up indicating absence of reinnervation. This case demonstrates that TTS could develop in a denervated heart, although another possibility is limited reinnervation, below the threshold of detection of the MIBG scan. It is also possible that the transplanted heart is hypersensitive to circulating catecholamines due to the up-regulation of β-adrenergic receptors, increasing susceptibility to TTS.

There have also been case reports of several variants of TTS in the same patient [78,79]. These cases contradict the hypothesis that the distribution of β receptors in the myocardium dictates the TTS variant. Other modifying factors may also determine the region of involvement [80]. At present, it is unclear whether the distribution of β receptors in the myocardium evolves during an individual’s lifetime. These observations indicate that TTS is a much more complex disorder than previously thought. TTS may be a combination of several heterogeneous conditions with multiple pathophysiology pathways, leading to a common phenotype. We can only hope that the answer to these questions will become more transparent with more focused future research.

### 4.7. Future Directions

Nuclear imaging has a significant role in evaluating, diagnosing, and prognosticating patients with TTS. At present, neither the Revised Mayo Clinic Criteria nor the criteria by the Takotsubo Cardiomyopathy Study Group (TCSG) incorporate nuclear imaging into the diagnostic algorithm for TTS [1,81]. Given the consistent presence of the ‘inverse-mismatch’ pattern of perfusion and metabolism in TTS, one could argue for modifying these diagnostic criteria to include nuclear imaging. If future studies show sufficient sensitivity and specificity for nuclear imaging in diagnosing TTS, one could potentially avoid invasive procedures such as angiography for its diagnosis. Moreover, as several studies have demonstrated, nuclear imaging studies provide excellent prognostic information in TTS. These arguments favor incorporating nuclear imaging studies into the TTS diagnostic algorithm. Finally, using nuclear imaging to evaluate neurobiological activity may help predict a person’s lifetime risk of TTS. This information could potentially be used to modify behavior and avoid situations that may precipitate TTS. As technology evolves further, and we are able to custom-design tracers targeting specific receptors in the myocardium, we will surely gain more insights into this fascinating disorder.

### 4.8. Limitations

Our review, although comprehensive, had several limitations. Firstly, only case reports, case series, and observational studies were included in this review. Additionally, the sample size of the observational studies was small. There was no uniform method of reporting results and imaging features, and most studies did not give additional information, such as coronary angiography or echocardiography findings. The use of stress studies was also unclear in the majority of the cases. Well-designed randomized controlled trials would better answer the questions that we have about the role of nuclear imaging in TTS. Until then, our best data are being collated from observational studies and anecdotal evidence.

## 5. Conclusions

TTS is a condition characterized by transient ventricular dysfunction. Nuclear imaging of TTS reveals perfusion defects in a minority of patients and metabolic defects in a majority. These defects do not conform to a unique epicardial coronary territory. Moreover, the metabolic defect is almost always out of proportion to the perfusion defect leading to a characteristic ‘inverse-mismatch’ pattern. Nuclear imaging offers the possibility to separately study myocardial perfusion, sympathetic innervation, glucose metabolism, fatty acid metabolism, and myocardial inflammation. In the future, nuclear imaging techniques may be adopted for risk prediction, stratification, and prognostication of TTS. However, more research is needed before nuclear imaging becomes mainstream in evaluating TTS.

## Figures and Tables

**Table 1:** Studies reporting nuclear imaging findings in Takotsubo cardiomyopathy.

Sl. No.	Author	Number of Patients	Age in Years, Sex	Perfusion Study	Perfusion Defect	Additional Tracer Study	Reduced Uptake of Additional Tracer	Perfusion-Metabolic Mismatch	Region Maximally Involved	Reversal of Imaging Findings
1	Anderson et al. [2]	16	Not reported	^99m^Tc sestamibi (MIBI) SPECT: 6, ^201^Tl SPECT: 2, ^82^Rb PET: 8	+ in 11/16 (68.7%)	Not performed	NR	NA	Apex in 9/11 abnormal studies	NR
2	Yao et al. [3]	1	75, M	^99m^Tc sestamibi (MIBI) SPECT	+	^18^F FDG PET	++	NR	Apex	NR
3	Albert et al. [4]	1	77, F	^82^Rb PET	+	^18^F FDG PET	+++	+	Apex	NR
4	Ito et al. [5]	1	62, F	^99m^Tc tetrofosmin SPECT	NR	^123^I BMIPP SPECT	NR	+	Midventricular circumference	NR
5	Sestini et al. [6]	22	70 +/− 11, F:21, M:1	^99m^Tc sestamibi (MIBI) SPECT	++ in 17/22 (77.2%)	^123^I MIBG SPECT	++	+	Apex	In few who underwent follow-up scans
6	Crimizade et al. [7]	1	84, F	^99m^Tc tetrofosmin SPECT	++	^123^I MIBG SPECT, ^18^F FDG PET	++	-	Apex	Yes, after 1 month
7	Nagai et al. [8]	1	74, F	^201^Tl SPECT	-	^123^I BMIPP SPECT	++	+	Mid-ventricle	NR
8	Humbert et al. [9]	1	41, F	Not performed	NA	^123^I MIBG SPECT	++	NA	Basal	NR
9	Messas et al. [10]	1	72, F	^99m^Tc tetrofosmin SPECT	-	^123^I MIBG SPECT	++	+	Apex	NR
10	Harris et al. [11]	1	45, F	^99m^Tc sestamibi (MIBI) SPECT	+	^123^I MIBG SPECT	++	+	Apex	Yes, after 6 weeks
11	Matsuo et al. [12]	16	72 +/− 3, 50% F	^99m^Tc sestamibi (MIBI) SPECT or ^99m^Tc tetrofosmin SPECT	++	^123^I BMIPP SPECT	++	+	Apex	NR
12	Ghadri et al. [13]	1	80, F	^13^N ammonia PET	++	^18^F FDG PET	++	-	Apex	Yes, after 3 months
13	Ikutomi et al. [14]	1	64. F	^99m^Tc sestamibi (MIBI) SPECT	-	^123^I MIBG SPECT	++	+	Apex	NR
14	Ibrahim et al. [15]	1	70, F	Not performed	NA	^18^F FDG PET	++	NA	Apex	NR
15	Arao et al. [16]	1	83, F	Not performed	NA	^123^I MIBG SPECT	++	NA	Mid-ventricle	NR
16	Miyachi et al. [17]	1	85, F	^99m^Tc sestamibi (MIBI) SPECT	+	^123^I BMIPP SPECT, 18F FDG PET (fasting protocol)	++	+	Apex	NR
17	Chrapko et al. [18]	1	46, F	^99m^Tc sestamibi (MIBI) SPECT	-	^123^I MIBG SPECT	++	+	Apex	NR
18	Arias et al. [19]	1	77, F	^99m^Tc sestamibi (MIBI) SPECT	++	Not performed	NR	NA	Apex	NR
19	Bonnemeier et al. [20]	37	68.6 +/− 11, 97% F	^99m^Tc sestamibi (MIBI) SPECT	-	^18^F FDG PET, ^123^I MIBG	++	+	Apex (27), Mid-ventricle(10)	NR
20	Cimarelli et al. [21]	18	67 +/− 22.6, 72% F	^99m^Tc tetrofosmin SPECT, ^201^Tl SPECT	-	^18^F FDG PET, ^123^I MIBG	+++	+	Apex (13), Mid-ventricle (5)	Yes, after 6 months in those who had follow- up scans
21	Skovgaard et al. [22]	1	72, F	^99m^Tc sestamibi (MIBI) SPECT	++	^18^F FDG PET, ^123^I MIBG	++	+	Apex	Yes, after 3 months
22	Soares-Filho et al. [23]	1	66, F	Not performed	NA	^123^I MIBG SPECT	++	NA	Apex	NR
23	Prasad et al. [24]	1	54, F	^13^N ammonia PET	-	^11^C hydroxy-ephedrine	++	+	Mid-ventricle	NR
24	Ishibashi et al. [25]	1	66, M	^201^Tl SPECT	+	^123^I BMIPP SPECT	+++	+	Apex	NR
25	Davis et al. [26]	1	49, M	^99m^Tc sestamibi (MIBI) SPECT	++	Not performed	NR	NA	Basal (reverse TTC)	NR
26	Morel et al. [27]	17	72.5 +/− 9, 100% F	^201^Tl SPECT, ^99m^Tc tetrofosmin SPECT	-	^18^F FDG PET, ^123^I MIBG SPECT	++	+	Apex	Yes, in few patients
27	Izumi et al. [28]	1	73, F	^201^Tl SPECT	+	^123^I MIBG SPECT	++	+	Apex	NR
28	Burgdorf et al. [29]	2	45,41; Both F	^99m^Tc sestamibi (MIBI) SPECT	-	^18^F FDG PET, ^123^I MIBG SPECT	+	NR	Apex	NR
29	Moriya et al. [30]	1	67, F	Not performed	NA	^123^I BMIPP SPECT, ^123^I-MIBG SPECT	++	NA	Mid-ventrice	No, at 6 months
30	Uchida et al. [31]	9	74 +/− 9.9; 77% F	^201^Tl SPECT, ^99m^Tc sestamibi (MIBI) SPECT	++	^123^I BMIPP SPECT, ^123^I MIBG SPECT	++	+	Apex	Yes, fully in one group, partially in the other
31	Feola et al. [32]	3	65, 74, 87; All F	^13^N ammonia PET	+	^18^F FDG PET	++	+	Apex	Yes, after 3 months
32	Izumi et al. [28]	1	73, F	^201^Tl SPECT	+	^123^I MIBG SPECT	++	+	Apex	NR
33	Cimarelli et al. [33]	2	85, 67; Both F	^99m^Tc tetrofosmin SPECT	-	^18^F FDG PET, ^123^I MIBG SPECT	++	+	Apex (1), Mid-ventricular (1)	NR
34	De Boeck et al. [34]	1	73, F	^99m^Tc tetrofosmin SPECT	++	^18^F FDG PET	+++	-	Apex	Yes, after 2 weeks
35	Burgdorf et al. [35]	10	67 +/− 4; 90% F	^99m^Tc sestamibi (MIBI) SPECT	+	^123^I MIBG SPECT	+++	+	Apex	NR
36	Yoshida et al. [36]	15	72 +/− 7; 80% F	^201^Tl SPECT	++	^18^F FDG PET	++	+	Apex	NR
37	Takeoka et al. [37]	2	73, 56, Both F	^201^Tl SPECT	-	^123^I MIBG SPECT	++	+	Apex	NR
38	Alexanderson et al. [38]	1	65, F	^99m^Tc sestamibi (MIBI) SPECT	++	Not performed	NR	NA	Apex	Yes, after one month
39	Rendl et al. [39]	1	67, F	^99m^Tc tetrofosmin SPECT	+	^18^F FDG PET	+++	+	Apex	Yes, after 27 days
40	Scholte et al. [40]	1	64, F	Not performed	NA	^123^I MIBG SPECT	++	NA	Apex	NR
41	Bybee et al. [41]	4	60, 66, 82, 75; All F	^13^N ammonia PET	++	^18^F FDG PET	++	+ in 50%	Apex	NR
42	Pessoa et al. [42]	5	67 +/− 14, All F	Not performed	NA	^67^Ga citrate, ^123^I MIBG SPECT	++	+	Apex	NR
43	Feola et al. [43]	1	65, F	^13^N ammonia PET	-	^18^F FDG PET	++	+	Apex	Yes
44	Sakuragi et al. [44]	1	59, F	^201^Tl SPECT	+	^123^I BMIPP SPECT, ^123^I MIBG SPECT	++	+	Apex	NR
45	Fukui et al. [45]	1	84, F	^99m^Tc sestamibi (MIBI) SPECT	-	^123^I BMIPP SPECT	++	+	Apex	Partial, after 3 months
46	Obunai et al. [46]	1	52, F	^82^Rb PET	++	^18^F FDG PET	++	+	Apex	Yes
47	Ohwada et al. [47]	3	17, 25, 33; All F	^201^Tl SPECT	-	^123^I BMIPP SPECT, ^123^I MIBG SPECT	++	+	Apex	NR
48	Suzuki et al. [48]	1	64, M	^201^Tl SPECT	-	^123^I BMIPP SPECT	++	+	Apex	NR
49	Miyazaki et al. [49]	1	79, F	^201^Tl SPECT	++	^123^I MIBG SPECT	++	-	Apex	Yes, after 3 months
50	Nishikawa et al. [50]	1	84, F	^99m^Tc tetrofosmin SPECT	++	^123^I BMIPP SPECT, ^123^I MIBG SPECT	++	-	Apex	Partial, after 14 days
51	Hadase et al. [51]	1	82, F	^99m^Tc sestamibi (MIBI) SPECT	++	Not performed	NR	NA	Apex	NR
52	Moriya et al. [30]	1	69, M	Not performed	NA	^123^I BMIPP SPECT, ^123^I MIBG SPECT	++	NA	Apex	Partial, after 3 months
53	Owa et al. [52]	4	70, 66, 53, 69; All F	^201^Tl SPECT	+	^123^I MIBG SPECT, ^123^I BMIPP SPECT	++	+	Apex	Partial

NR: Not reported, NA: Not applicable, +: Mild reduction in additional tracer uptake, ++: Moderate to severe reduction in additional tracer uptake, +++: Absent additional tracer uptake.

## References

[1] Prasad A., Lerman A., Rihal C.S. (2008). Apical Ballooning Syndrome (Tako-Tsubo or Stress Cardiomyopathy): A Mimic of Acute Myocardial Infarction. Am. Heart J..

[2] Anderson J.L., Horne B.D., Le V.T., Bair T.L., Min D.B., Minder C.M., Dhar R., Mason S., Muhlestein J.B., Knowlton K.U. (2020). Spectrum of Radionuclide Perfusion Study Abnormalities in Takotsubo Cardiomyopathy. J. Nucl. Cardiol..

[3] Yao Z., Hu J., Yan Y., Ge J. (2019). Specific Manifestation of Single-Photon Emission Computed Tomography and Positron Emission Tomography Magnetic Resonance Imaging in a Man With Takotsubo Cardiomyopathy. Circ. Cardiovasc. Imaging.

[4] Albert C.L., White K.T., Cremer P.C., Jaber W.A. (2019). Stress for a Stressed Out Heart: Classic Cardiac PET Findings in Takotsubo Cardiomyopathy. J. Nucl. Cardiol..

[5] Ito S., Endo A., Morita Y., Tanabe K. (2018). Midventricular Takotsubo Cardiomyopathy on 99mTc-tetrofosmin and 123i-BMIPP SPECT. Intern. Med..

[6] Sestini S., Pestelli F., Leoncini M., Bellandi F., Mazzeo C., Mansi L., Carrio I., Castagnoli A. (2017). The Natural History of Takotsubo Syndrome: A Two-Year Follow-up Study with Myocardial Sympathetic and Perfusion G-SPECT Imaging. Eur. J. Nucl. Med. Mol. Imaging.

[7] Crimizade U., Messas N., Blondet C., Jesel L., Duculescu E., Ohlmann P., Morel O. (2016). Biventricular Takotsubo Cardiomyopathy Triggered by Myocardial Ischemic Injury: Insights from Multimodal Imaging Approach. Int. J. Cardiol..

[8] Nagai T., Konishi T., Arakawa J., Hisadome H., Tabata H. (2014). Synchronicity of Echocardiography and Cardiac Nuclear Medicine in Mid-Ventricular Ballooning Syndrome: Paired ’Ring Signs’ on Polar Maps. Eur. Heart J. Cardiovasc. Imaging.

[9] Humbert O., Stamboul K., Gudjoncik A., Kanoun S., Richard C., Cochet A., Cottin Y. (2015). Dual Diagnostic Role of 123i-MIBG Scintigraphy in Inverted-Takotsubo Pattern Cardiomyopathy. Clin. Nucl. Med..

[10] Messas N., Blondet C., Jesel L., Hess S., Girardey M., Imperiale A., Khouri T., Ohlmann P., Morel O. (2015). Diagnostic Relevance of Optical Coherence Tomography Imaging in Aborted Acute Myocardial Infarction with a “Takotsubo Component”. Int. J. Cardiol..

[11] Harris D., Wong T.C., Soman P. (2015). Direct Visualization of Regional Cardiac Sympathetic Dysfunction in Stress-Induced Cardiomyopathy. J. Nucl. Cardiol..

[12] Matsuo S., Nakajima K., Kinuya S., Yamagishi M. (2014). Diagnostic Utility of 123i-BMIPP Imaging in Patients with Takotsubo Cardiomyopathy. J. Cardiol..

[13] Ghadri J.R., Dougoud S., Maier W., Kaufmann P.A., Gaemperli O., Prasad A., Lüscher T.F., Templin C. (2014). A PET/CT-follow-up Imaging Study to Differentiate Takotsubo Cardiomyopathy from Acute Myocardial Infarction. Int. J. Cardiovasc. Imaging.

[14] Ikutomi M., Yamasaki M., Matsusita M., Watari Y., Arashi H., Endo G., Yamaguchi J., Ohnishi S. (2014). Takotsubo Cardiomyopathy in Siblings. Heart Vessel..

[15] Ibrahim T., Nekolla S.G., Langwieser N., Rischpler C., Groha P., Laugwitz K.-L., Schwaiger M. (2012). Simultaneous Positron Emission Tomography/Magnetic Resonance Imaging Identifies Sustained Regional Abnormalities in Cardiac Metabolism and Function in Stress-Induced Transient Midventricular Ballooning Syndrome: A Variant of Takotsubo Cardiomyopathy. Circulation.

[16] Arao K., Ako J., Momomura S. (2013). Transient Mid-Ventricular Ballooning: Insights from 123I-metaiodobenzylguanidine (MIBG) Scintigraphy. Int. J. Cardiol..

[17] Miyachi H., Kumita S., Tanaka K. (2013). PET/CT and SPECT/CT Cardiac Fusion Imaging in a Patient with Takotsubo Cardiomyopathy. Eur. Heart J..

[18] Chrapko B.E., Tomaszewski A., Jaroszyński A.J., Furmaga J., Wysokiński A., Rudzki S. (2012). Takotsubo Syndrome in a Patient After Renal Transplantation. Med. Sci. Monit..

[19] Arias A.M., Oberti P.F., Pizarro R., Falconi M.L., de Arenaza D.P., Zeffiro S., Cagide A.M. (2011). Dobutamine-Precipitated Takotsubo Cardiomyopathy Mimicking Acute Myocardial Infarction: A Multimodality Image Approach. Circulation.

[20] Bonnemeier H., Demming T., Weidtmann B., Ortak J., Burgdorf C., Reppel M., Mäuser W., Rosenberg M., Frey N. (2010). Differential Heart Rate Dynamics in Transient Left Ventricular Apical and Midventricular Ballooning. Heart Rhythm..

[21] Cimarelli S., Sauer F., Morel O., Ohlmann P., Constantinesco A., Imperiale A. (2010). Transient Left Ventricular Dysfunction Syndrome: Patho-Physiological Bases Through Nuclear Medicine Imaging. Int. J. Cardiol..

[22] Skovgaard D., Holmvang L., Bang L.E., Lønborg J., Hasbak P. (2010). Imaging of Takotsubo Cardiomyopathy. Clin. Nucl. Med..

[23] Soares-Filho G.L., Felix R.C., Azevedo J.C., Mesquita C.T., Mesquita E.T., Valença A.M., Nardi A.E. (2010). Broken Heart or Takotsubo Syndrome: Support for the Neurohumoral Hypothesis of Stress Cardiomyopathy. Prog. Neuropsychopharmacol. Biol. Psychiatry.

[24] Prasad A., Madhavan M., Chareonthaitawee P. (2009). Cardiac Sympathetic Activity in Stress-Induced (Takotsubo) Cardiomyopathy. Nat. Rev. Cardiol..

[25] Ishibashi K., Osamura T., Yamahara Y. (2009). Myocardial Stunning with Partial Aneurysmal Formation Generated during the Recovering Process of Tachycardia-Induced Cardiomyopathy. J. Cardiol..

[26] Davis M., Hardebeck C. (2009). Reverse Takotsubo Syndrome Diagnosed with Tc-99m SPECT Perfusion Study. J. Nucl. Cardiol..

[27] Morel O., Sauer F., Imperiale A., Cimarelli S., Blondet C., Jesel L., Trinh A., De Poli F., Ohlmann P., Constantinesco A. (2009). Importance of Inflammation and Neurohumoral Activation in Takotsubo Cardiomyopathy. J. Card. Fail..

[28] Izumi K., Tada S., Yamada T. (2008). A Case of Takotsubo Cardiomyopathy Complicated by Ventricular Septal Perforation. Circ. J..

[29] Burgdorf C., Bonnemeier H., Vonhof K., Schunkert H., Kurowski V. (2008). Coronary Artery Vasospasm or True Transient Left Ventricular Apical Ballooning? Differentiation by Nuclear Imaging. J. Nucl. Cardiol..

[30] Moriya M., Mori H., Suzuki N., Hazama M., Yano K. (2002). Six-Month Follow-up of Takotsubo Cardiomyopathy with I-123-beta-metyl-iodophenyl Pentadecanoic Acid and I-123-meta-iodobenzyl-guanidine Myocardial Scintigraphy. Intern. Med..

[31] Uchida Y., Nanjo S., Fujimoto S., Yamashina S., Wagatsma K., Nakano H., Yamazaki J. (2008). Scintigraphic Studies on the Etiology of Ampulla Cardiomyopathy. J. Cardiol..

[32] Feola M., Chauvie S., Rosso G.L., Biggi A., Ribichini F., Bobbio M. (2008). Reversible Impairment of Coronary Flow Reserve in Takotsubo Cardiomyopathy: A Myocardial PET Study. J. Nucl. Cardiol..

[33] Cimarelli S., Imperiale A., Ben-Sellem D., Rischner J., Detour J., Morel O., Ohlmann P., Constantinesco A. (2008). Nuclear Medicine Imaging of Takotsubo Cardiomyopathy: Typical Form and Midventricular Ballooning Syndrome. J. Nucl. Cardiol..

[34] De Boeck B.W.L., Verburg F.A., Hobbelink M.G.G., Velthuis B., Melman P.G., Cramer M.-J.M. (2008). Reversible 18-FDG-uptake Defects on Myocardial PET: Is This Myocardial Resurrection?. Int. J. Cardiol..

[35] Burgdorf C., von Hof K., Schunkert H., Kurowski V. (2008). Regional Alterations in Myocardial Sympathetic Innervation in Patients with Transient Left-Ventricular Apical Ballooning (Tako-Tsubo Cardiomyopathy). J. Nucl. Cardiol..

[36] Yoshida T., Hibino T., Kako N., Murai S., Oguri M., Kato K., Yajima K., Ohte N., Yokoi K., Kimura G. (2008). A Pathophysiologic Study of Tako-Tsubo Cardiomyopathy with F-18 Fluorodeoxyglucose Positron Emission Tomography. Eur. Heart J..

[37] Takeoka Y., Nakamae M., Nakamae H., Hagihara K., Sakamoto E., Nakane T., Koh H., Koh K.-R., Ohta K., Yamane T. (2007). Two Cases of Ampulla (Takotsubo-Shaped) Cardiomyopathy Associated with Hemophagocytic Lymphohistiocytosis. Acta Haematol..

[38] Alexanderson E., Cruz P., Talayero J., Damas F., Zeron J., Meave A. (2007). Transient Perfusion and Motion Abnormalities in Takotsubo Cardiomyopathy. J. Nucl. Cardiol..

[39] Rendl G., Rendl G., Altenberger J., Pirich C. (2006). Takotsubo Cardiomyopathy in Positron Emission Tomography. Wien. Klin. Wochenschr..

[40] Scholte A., Bax J., Stokkel M., Plokker T., Kaandorp T., Lamb H., Deroos A., Vanderwall E. (2006). Multimodality Imaging to Diagnose Takotsubo Cardiomyopathy. J. Nucl. Cardiol..

[41] Bybee K., Murphy J., Prasad A., Wright R., Lerman A., Rihal C., Chareonthaitawee P. (2006). Acute Impairment of Regional Myocardial Glucose Uptake in the Apical Ballooning (Takotsubo) Syndrome. J. Nucl. Cardiol..

[42] Pessoa P.M.C., Xavier S.S., Lima S.L.R., Mansur J., de Almeida A.S., Carvalho P.A.C., Gutfilen B., da Fonseca B.L.M. (2006). Assessment of Takotsubo (Ampulla) Cardiomyopathy Using Iodine-123 Metaiodobenzylguanidine Scintigraphy. Acta Radiol..

[43] Feola M., Rosso G., Casasso F., Morena L., Biggi A., Chauvie S., Ribichini F., Uslenghi E. (2006). Reversible Inverse Mismatch in Transient Left Ventricular Apical Ballooning: Perfusion/Metabolism Positron Emission Tomography Imaging. J. Nucl. Cardiol..

[44] Sakuragi S., Tokunaga N., Okawa K., Kakishita M., Ohe T. (2007). A Case of Takotsubo Cardiomyopathy Associated with Epileptic Seizure: Reversible Left Ventricular Wall Motion Abnormality and ST-segment Elevation. Heart Vessel..

[45] Fukui M., Mori Y., Tsujimoto S., Takehana K., Sakamoto N., Kishimoto N., Imada T., Maeba H., Nose A., Yamahara H. (2006). “Takotsubo” Cardiomyopathy in a Maintenance Hemodialysis Patient: Takotsubo Cardiomyopathy and Hemodialysis. Ther. Apher. Dial..

[46] Obunai K., Misra D., Vantosh A., Bergmann S. (2005). Metabolic Evidence of Myocardial Stunning in Takotsubo Cardiomyopathy: A Positron Emission Tomography Study. J. Nucl. Cardiol..

[47] Ohwada R., Hotta M., Kimura H., Takagi S., Matsuda N., Nomura K., Takano K. (2005). Ampulla Cardiomyopathy after Hypoglycemia in Three Young Female Patients with Anorexia Nervosa. Intern. Med..

[48] Suzuki K., Osada N., Akasi Y.J., Suzuki N., Sakakibara M., Miyake F., Maki F., Takahashi Y. (2004). An Atypical Case of “Takotsubo Cardiomyopathy” during Alcohol Withdrawal: Abnormality in the Transient Left Ventricular Wall Motion and a Remarkable Elevation in the ST Segment. Intern. Med..

[49] Miyazaki S., Kamiishi T., Hosokawa N., Komura M., Konagai H., Sagai H., Takamoto T. (2004). Reversible Left Ventricular Dysfunction “Takotsubo” Cardiomyopathy Associated with Hyperthyroidism. Jpn. Heart J..

[50] Nishikawa S., Ito K., Adachi Y., Katoh S., Azuma A., Matsubara H. (2004). Ampulla (‘Takotsubo’) Cardiomyopathy of Both Ventricles: Evaluation of Microcirculation Disturbance Using 99mTc-tetrofosmin Myocardial Single Photon Emission Computed Tomography and Doppler Guide Wire. Circ. J..

[51] Hadase M., Kawasaki T., Asada S., Kamitani T., Kawasaki S., Sugihara H. (2003). Reverse Redistribution of Tc-99m Tetrofosmin in a Patient with “Takotsubo” Cardiomyopathy. Clin. Nucl. Med..

[52] Owa M., Aizawa K., Urasawa N., Ichinose H., Yamamoto K., Karasawa K., Kagoshima M., Koyama J., Ikeda S. (2001). Emotional Stress-Induced “Ampulla Cardiomyopathy”: Discrepancy Between the Metabolic and Sympathetic Innervation Imaging Performed during the Recovery Course. Jpn. Circ. J..

[53] John K., Lal A., Mishra A. (2021). A Review of the Presentation and Outcome of Takotsubo Cardiomyopathy in COVID-19. Monaldi Arch. Chest Dis..

[54] Sato M., Fujita S., Saito A., Ikeda Y., Kitazawa H., Takahashi M., Ishiguro J., Okabe M., Nakamura Y., Nagai T. (2006). Increased Incidence of Transient Left Ventricular Apical Ballooning (So-Called Takotsubo’ Cardiomyopathy) after the Mid-Niigata Prefecture Earthquake. Circ. J..

[55] Ellison G.M., Torella D., Karakikes I., Purushothaman S., Curcio A., Gasparri C., Indolfi C., Cable N.T., Goldspink D.F., Nadal-Ginard B. (2007). Acute β-Adrenergic Overload Produces Myocyte Damage Through Calcium Leakage from the Ryanodine Receptor 2 but Spares Cardiac Stem Cells. J. Biol. Chem..

[56] Lyon A.R., Rees P.S., Prasad S., Poole-Wilson P.A., Harding S.E. (2008). Stress (Takotsubo) Cardiomyopathy—A Novel Pathophysiological Hypothesis to Explain Catecholamine-Induced Acute Myocardial Stunning. Nat. Rev. Cardiol..

[57] Verschure D.O., Somsen G.A., van Eck-Smit B.L.F., Knol R.J.J., Booij J., Verberne H.J. (2014). Tako-Tsubo Cardiomyopathy: How to Understand Possible Pathophysiological Mechanism and the Role of (123)I-MIBG Imaging. J. Nucl. Cardiol..

[58] Paur H., Wright P.T., Sikkel M.B., Tranter M.H., Mansfield C., O’Gara P., Stuckey D.J., Nikolaev V.O., Diakonov I., Pannell L.M.K. (2012). High Levels of Circulating Epinephrine Trigger Apical Cardiodepression in a Β2 Adrenergic Receptor Gi Dependent Manner a New Model of Takotsubo Cardiomyopathy. Circulation.

[59] Kawano H., Okada R., Yano K. (2003). Histological Study on the Distribution of Autonomic Nerves in the Human Heart. Heart Vessel..

[60] Christensen T.E., Bang L.E., Holmvang L., Skovgaard D.C., Oturai D.B., Søholm H., Thomsen J.H., Andersson H.B., Ghotbi A.A., Ihlemann N. (2016). (123)I-MIBG Scintigraphy in the Subacute State of Takotsubo Cardiomyopathy. JACC Cardiovasc. Imaging.

[61] Malhotra S., Fernandez S.F., Fallavollita J.A., Canty J.M. (2015). Prognostic Significance of Imaging Myocardial Sympathetic Innervation. Curr. Cardiol. Rep..

[62] Matsuura T., Ueno M., Iwanaga Y., Miyazaki S. (2019). Importance of Sympathetic Nervous System Activity during Left Ventricular Functional Recovery and Its Association with in-Hospital Complications in Takotsubo Syndrome. Heart Vessel..

[63] Burger I.A., Lohmann C., Messerli M., Bengs S., Becker A.S., Maredziak M., Treyer V., Haider A., Schwyzer M., Benz D.C. (2018). Age- and Sex-Dependent Changes in Sympathetic Activity of the Left Ventricular Apex Assessed by 18f-DOPA PET Imaging. PLoS ONE.

[64] Christensen T.E., Ahtarovski K.A., Bang L.E., Holmvang L., Søholm H., Ghotbi A.A., Andersson H., Vejlstrup N., Ihlemann N., Engstrøm T. (2015). Basal Hyperaemia Is the Primary Abnormality of Perfusion in Takotsubo Cardiomyopathy: A Quantitative Cardiac Perfusion Positron Emission Tomography Study. Eur. J. Echocardiogr..

[65] Kobylecka M., Budnik M., Kochanowski J., Piątkowski R., Wojtera K., Chojnowski M., Peller M., Fronczewska-Wieniawska K., Mazurek T., Mączewska J. (2019). Diagnostic Utility of Hybrid Single Photon Emission Computed Tomography/Computed Tomography Imaging in Patients with Takotsubo Syndrome. J. Cardiovasc. Med. Hagerstown.

[66] Sugihara Y., Fukushima Y., Kumita S.-I., Takano H., Shimizu W. (2018). Diagnostic Performance of Hybrid Cardiac SPECT/CT Imaging for Patients with Takotsubo Cardiomyopathy. Eur. J. Hybrid Imaging.

[67] Kurisu S., Inoue I., Kawagoe T., Ishihara M., Shimatani Y., Nishioka K., Umemura T., Nakamura S., Yoshida M., Sato H. (2003). Myocardial Perfusion and Fatty Acid Metabolism in Patients with Tako-Tsubo-Like Left Ventricular Dysfunction. J. Am. Coll. Cardiol..

[68] Kobylecka M., Budnik M., Kochanowski J., Piatkowski R., Chojnowski M., Fronczewska-Wieniawska K., Mazurek T., Maczewska J., Peller M., Opolski G. (2018). Takotsubo Cardiomyopathy: FDG Myocardial Uptake Pattern in Fasting Patients. Comparison of PET/CT, SPECT, and ECHO Results. J. Nucl. Cardiol..

[69] Paolisso P., Foà A., Bergamaschi L., Donati F., Fabrizio M., Chiti C., Angeli F., Toniolo S., Stefanizzi A., Armillotta M. (2021). Hyperglycemia, Inflammatory Response and Infarct Size in Obstructive Acute Myocardial Infarction and MINOCA. Cardiovasc. Diabetol..

[70] Paolisso P., Bergamaschi L., Rambaldi P., Gatta G., Foà A., Angeli F., Fabrizio M., Casella G., Barbieri M., Galiè N. (2021). Impact of Admission Hyperglycemia on Heart Failure Events and Mortality in Patients with Takotsubo Syndrome at Long-term Follow-up: Data from HIGH-GLUCOTAKO Investigators. Diabetes Care.

[71] Suzuki H., Matsumoto Y., Kaneta T., Sugimura K., Takahashi J., Fukumoto Y., Takahashi S., Shimokawa H. (2014). Evidence for Brain Activation in Patients with Takotsubo Cardiomyopathy. Circ. J..

[72] Tawakol A., Ishai A., Takx R.A., Figueroa A.L., Ali A., Kaiser Y., Truong Q.A., Solomon C.J., Calcagno C., Mani V. (2017). Relation between Resting Amygdalar Activity and Cardiovascular Events: A Longitudinal and Cohort Study. Lancet.

[73] Templin C., Hänggi J., Klein C., Topka M.S., Hiestand T., Levinson R.A., Jurisic S., Lüscher T.F., Ghadri J.-R., Jäncke L. (2019). Altered Limbic and Autonomic Processing Supports Brain-Heart Axis in Takotsubo Syndrome. Eur. Heart J..

[74] Radfar A., Abohashem S., Osborne M.T., Wang Y., Dar T., Hassan M.Z.O., Ghoneem A., Naddaf N., Patrich T., Abbasi T. (2021). Stress-Associated Neurobiological Activity Associates with the Risk for and Timing of Subsequent Takotsubo Syndrome. Eur. Heart J..

[75] Suzuki H., Takanami K., Takase K., Shimokawa H., Yasuda S. (2021). Reversible Increase in Stress-Associated Neurobiological Activity in the Acute Phase of Takotsubo Syndrome; a Brain 18f-FDG-PET Study. Int. J. Cardiol..

[76] Miyake R., Ohtani K., Hashimoto T., Yada R., Sato T., Shojima Y., Hayashidani S., Higo T., Tsutsui H. (2020). Takotsubo Syndrome in a Heart Transplant Recipient with Poor Cardiac Sympathetic Reinnervation. ESC Heart Fail..

[77] Beckers F., Ramaekers D., Speijer G., Ector H., Vanhaecke J., Verheyden B., Van Cleemput J., Droogné W., Van de Werf F., Aubert A.E. (2004). Different Evolutions in Heart Rate Variability after Heart Transplantation: 10-Year Follow-Up. Transplantation.

[78] Ghadri J.R., Jaguszewski M., Corti R., Lüscher T.F., Templin C. (2012). Different Wall Motion Patterns of Three Consecutive Episodes of Takotsubo Cardiomyopathy in the Same Patient. Int. J. Cardiol..

[79] Frankel E., Prior P., Mehta H., Rajagopalan P., Wiener D.H., Rose A., Marhefka G.D. (2020). One Patient, Three Variants, Four Episodes of Takotsubo Cardiomyopathy. CASE.

[80] George A.A., John K.J., Selvaraj V., Mishra A.K. (2021). Endocrinological Abnormalities and Takotsubo Cardiomyopathy. Monaldi Arch. Chest Dis..

[81] Kawai S., Kitabatake A., Tomoike H. (2007). Takotsubo Cardiomyopathy Study Group Guidelines for Diagnosis of Takotsubo (Ampulla) Cardiomyopathy. Circ. J..

